# 

**Published:** 1973-10

**Authors:** 


Bristol Medico-Chirurgical Journal

(Incorporating the Medical Journal of the South-West)

Established 1883

Vol. 88 (iiii) OCTOBER 1973 No. 328

**Editor: N. J. Brown, M.B., F.R.C.P., F.R.C.Path.**

**Published Quarterly** **Annual Subscription £2 Post Free**

### BRISTOL MEDICO-CHIRURGICAL SOCIETY

**President 1972 - 73 :** Dr. James Macrae

**President-elect :** Dr. R. F. Barbour

**Honorary Secretary :** Dr. I. S. Bailey, 7 Percival Road, Bristol 8

**Honorary Treasurer :** Dr. Frank Ross, Bristol Royal Infirmary, Bristol 2

The Society was founded in 1874. In 1883 publication of the Journal began and has continued quarterly ever since then. During the years 1949 to 1962 it appeared under the title of "The Medical Journal of the South West".

### THE BRISTOL MEDICO-CHIRURGICAL JOURNAL

### Editorial Committee

| | |
|---|---|
| Editor | Dr. N. J. Brown, Southmead Hospital, Bristol. |
| Assistant Editor | Dr. D. S. Reeves. |
| Members | Dr. Rhys Davies.<br>Dr. M. B. Lennard.<br>Professor R. C. Wofinden.<br>Dr. D. W. Wright.<br>The Hon. Treasurer and Hon. Secretary of the Society. |
| Business Manager | Dr. P. J. F. Baskett, Frenchay Hospital, Bristol. |
| Secretary | Mr. B. P. Jones, The Library, Medical School, University Walk, Bristol BS8 1TD. |

### NOTICE TO CONTRIBUTORS

The Journal is especially concerned to provide a place for the recording of medical thought, interests, and practice in and around Bristol. It therefore welcomes articles that, as well as being of immediate interest to members, will document the local and conemporary medical scene for our successors.

Original articles are invited provided that they have not been published elsewhere. References in the text should be by author's name, with year of publication in parenthesis; they should be listed at the end in alphabetical order, the name of each journal or book being given in full—not abbreviated—and the title of the article added. Illustrations should be supplied ready for reproduction. Line drawing should be in Indian ink on firm white paper or board. The author will be informed if it is found necessary to ask him to pay for the making of blocks.

The following contributions will be especially welcome: notes of forthcoming events, meetings held, degrees conferred, staff appointments, honours and public appointments, and other local news.

### The Medical Library

In 1890 the Bristol Medico-Chirurgical Society founded a medical library which in 1892 was combined with that of the Bristol Medical School. This became the University of Bristol Medical Library in 1925 and an agreement in that year confirmed the privilege of Members of the Society to use the University Medical Library.

### A Selection of Recent Additions to the Medical Library

AAS, K.: Biochemical and immunological basis of chronic asthma. (Thomas).

AMERICAN ACADEMY OF ORTHOPEDIC SURGEONS.: Symposium on myelomeningocele. (Mosby).

ARIENS, E. J. ed.: Drug design. 3 vols. (Acad. Press).

ASHER, R.: Richard Asher talking sense. Ed. Sir Francis Avery Jones. (Pitman).

ASHLEY, D. J. B.: An introduction to the general pathology of tumours. (Wright). Presented by John Wright & Sons Ltd.

BAIRD, H. W.: The child with convulsions. (Grune & Stratton).

BARKER, D. J. P.: Practical epidemiology. (Churchill Livingstone).

BEHBEHANI, A. M.: Human viral, bedsonial and rickettsial diseases: a dianostic handbook for physicians. (Thomas).

BELINKOFF, S.: Emphysema and chronic bronchitis. (Little, Brown).

BELLET, S.: Essentials of cardiac arrhythmias: diagnosis and management. (Saunders).

BIRKMAYER, W. ed.: Spasticity: a topical survey. (Huber). Presented by CIBA Laboratories.

BLICKMAN, J. R. et al. eds.: Radiology. (Excerpta Medica).

BOERING, G.: Diseases of the oral cavity and salivary glands. (Wright). Presented by John Wright & Sons Ltd.

BOUCHIER, I. A. D.: Gastroenterology. (Baillière).

BRETLAND, P. M.: Acute ureteric obstruction: a clinical and radiological study (Butterworths).

BUECHNER, H. A. ed.: Management of fugus diseases of the lungs. (Thomas).

BURDETTE, W. J. & E. A. GEHAN.: Planning and analysis of clinical studies. (Thomas).

CAMERON, D. & L. M. MACBEAN.: A clinical study of infectious mononucleosis and toxoplasmosis. (Wright). Presented by John Wright & Sons Ltd.

CARSON, J.: X-ray diagnosis positioning manual. (Collier-Macmillan).

CHAPCHAL, G.: Operative treatment of scoliosis. (Thieme).

COHEN, J. O. ed.: The staphylococci. (Wiley).

COLE, R. B.: Essentials of respiratory disease. (Pitman).

CREMIN, B. J. et al.: Radiological diagnosis of digestive tract disorders in the newborn. (Butterworths).

CRITCHLEY, M. et al. eds.: Scientific foundations of neurology. (Heinemann).

CUMMING, G. & S. J. G. SEMPLE.: Disorders of the respiratory system. (Blackwell).

D'ARCY, P. F. & J. P. GRIFFIN: Iatrogenic diseases. (Oxford Univ. Press).

DAVIES, I. J. T.: The clinical significance of the essential biological metals. (Heinemann).

DAY, B. H.: Orthopaedic appliances. (Faber).

DICK, W. C.: Introduction to clinical rheumatology. (Churchill Livingstone).

DURANT, J. R. & R. V. SMALLEY.: The chronic leukaemias: chemistry, pathophysiology and treatment. (Thomas).

DUTHIE, R. B. et al.: The management of musculo-skeletal problems in the haemophilias. (Blackwell).

EDWARDS, C. H.: Neurology of ear, nose and throat diseases. (Butterworths).

ELDER, A. T. & D. W. NEILL eds.: Biomedical technology in hospital diagnosis. (Pergamon).

ELLIOTT, H. & K. RYZ.: Venereal diseases: treatment and nursing. (Baillière).

EMERY, A. E. H.: Antenatal diagnosis of genetic disease. (Churchill Livingstone).

FINK, B. R. ed.: Cellular biology and toxicity of anaesthetics. (Williams & Wilkins).

FIORENTINO, M. ed.: Newer anticancer drugs and procedures. (Piccin Med. Bks.).

FORREST, A. ed.: Companion to psychiatric studies. 2 vols. (Churchill Livingstone).

FOX, H. & F. A. LANGLEY eds.: Postgraduate obstetrical and gynaecological pathology. (Pergamon).

FROHLICH, E. D. ed.: Pathophysiology: altered regulatory mechanisms in disease. (Blackwell).

GAITE, C. M. ed.: Aging and the brain. (Plenum).

GARREY, M. M. et al.: Gynaecology illustrated. (Churchill Livingstone).

GILLESPIE, I. E. & T. J. THOMSON eds.: Gastroenterology: an integrated course. (Churchill Livingstone).

GLASS, D. C. & J. E. SINGER.: Urban stress: experiments on noise and social stressors. (Acad. Press).

### News and Notes

**NEW CONSULTANT APPOINTMENTS**

The following appointments have been made by the South-Western Regional Hospital Board.

A. R. R. Cain: Consultant Paediatrician, Bath Clinical Area.

C. D. Collins: Consultant General Surgeon, South Somerset Clinical Area.

E. J. Dabbs: Consultant Psychiatrist with a special interest in Rehabilitation, Bristol Clinical Area.

H. D. Fairman: Consultant E.N.T. Surgeon, Cornwall Clinical Area.

M. A. Hannon: Consultant Orthopaedic and Traumatic Surgeon, Bristol Clinical Area.

J. V. Lever: Consultant in Morbid Anatomy and Histopathology, Bath Clinical Area.

M. Messervy: Consultant E.N.T. Surgeon, Devon and Exeter Clinical Area, Torquay.

R. A. Seagger: Consultant Anaesthetist, Bath Clinical Area.

Dr. Henry Yellowlees, who was a member of the staff of the South-western Regional Hospital Board from 1954 to 1963, is to succeed Sir George Godber as Chief Medical Officer at the Department of Health and Social Security at the end of November. Dr. Yellowlees has been Deputy Chief Medical Officer since 1967.

---

During the spring of 1973, Dr. Beryl Corner spent three months in South East Asia as Consultant for the World Health Organisation advising on the practice and development of the eaching of neonatology. She visited medical colleges in Burma, Ceylon, India, Indonesia and Thailand.

---

In June 1973 the Faculty of Radiologists, of which Professor Middlemiss is President, held its Annual General Meeting in Bristol. There was a full scientific programme and the number of radiologists attending was the largest ever recorded at an Annual Meeting of the Faculty.

**INDEX TO VOLUME 88**

| AUTHORS | Page |
|---|---|
| Briggs, J. C. | 40 |
| Burston, G. R. | 35 |
| Evans, M. R. | 17 |
| Hamblin, T. J. | 11 |
| Hancock, C. C. | 51 |
| Jones, J. V. | 11 |
| Keatinge, W. R. | 39 |
| Macrae, J. | 23 |
| Morgans, C. C. | 1 |
| Nelson, S. D. | 31 |
| Preece, A. W. | 17 |
| White, H. J. O. | 31 |

| SUBJECTS | |
|---|---|
| Cold water, hazards | 39 |
| Geriatrics, community | 35 |
| Hospital, Bristol's fourth major | 40 |
| Jenner, Edward | 23 |
| Leukaemia, adult, acute | 11 |
| Maternity services in Bristol | 51 |
| Medical library | 21, 29, 34, 57 |
| News and notes | 30, 48, 58 |
| Organ matching and distribution | 31 |
| Saint Peter (The Perfectionist) | 1 |
| Society reports | 30, 42, 45, 46, 47 |
| Viscum album, possible cancer treatment | 17 |

